# Submerged Macrophytes Exhibit Different Phosphorus Stoichiometric Homeostasis

**DOI:** 10.3389/fpls.2018.01207

**Published:** 2018-08-14

**Authors:** Wei Li, Yujie Li, Jiayou Zhong, Hui Fu, Jie Tu, Houbao Fan

**Affiliations:** ^1^Jiangxi Provincial Engineering Research Center of Water Engineering Safety and Resources Efficient Utilization, Nanchang Institute of Technology, Nanchang, China; ^2^Ministry of Water Resources Research Center of Poyang Lake Water Resources and Water Environment, Jiangxi Institute of Water Sciences, Nanchang, China

**Keywords:** submersed macrophyte, phosphorus, ecological stoichiometry, interspecific difference, nutrient

## Abstract

Phosphorus (P) is a limiting element in many aquatic ecosystems. Excessive P input often leads to cyanobacterial bloom, thus triggering ecological imbalances and a series of environmental problems. Submerged macrophytes have a strong ability to absorb P and play important roles in maintaining aquatic ecosystem functions. However, the degree to which submerged macrophytes maintain their tissue P contents in various nutrient levels and the corresponding influencing factors are still not very clear. In this study, the stoichiometric characteristics and stoichiometric homeostasis of P in the aboveground and belowground parts of three submerged macrophytes, *Vallisneria natans* (Lour.) Hara, *Hydrilla verticillata* (L.f.) Royle, and *Ceratophyllum demersum* (L.), with great differences in growth forms, were studied under different growth times and nutrient levels via laboratory experiments. The results showed that the water conductivity, turbidity, and chlorophyll content increased significantly with the increasing nutrient levels. The variation of species, organ, growth time, and nutrient level could significantly affect the P contents of submerged macrophytes. Among these factors, the variance contribution rates caused by the differences of nutrient levels in water column were the highest at more than 50%. The P stoichiometric homeostasis index (*H*_P_) in the belowground parts of the three submerged macrophytes was higher than that of the aboveground parts. The *H*_P_ decreased by the growth time; the *H*_P_ of *V. natans* was significantly higher than those of *H. verticillata* and *C. demersum*. In summary, the P stoichiometric homeostasis in submerged macrophytes could reflect their responses to environmental changes, and the P content of submerged macrophytes was an indicator of the bioavailability of external P. *H. verticillata* exhibited a high growth rate and a high accumulation of P content, making it the most suitable species in this study for removing large amounts of P from water in a short term.

## Introduction

Ecological stoichiometric homeostasis refers to the ability of organisms to maintain the stability of their own element contents and ratios in a changing environment (Elser et al., 2000; Sterner and Elser, 2002; Yu et al., 2010; Yu et al., 2011). It is a basic theory of ecological stoichiometry and reflects the response of physiological and biochemical allocations within the organisms to the external environment (Yu et al., 2011; Leal et al., 2017). Yu et al. (2010) demonstrated that the stoichiometric homeostasis of Inner Mongolia grassland plants was positively correlated with its dominance and productivity in ecosystems, and plant communities with a high level of homeostasis also had a highly stable structure and function. The study by Gu et al. (2017) also showed that the phosphorus (P) and nitrogen (N): P stoichiometric homeostasis indices (*H*_P_’s) of tundra plants were positively correlated with the biomass of their aboveground parts.

Submerged macrophytes are important primary producers in aquatic ecosystems and they play an important role in maintaining the biological diversity and functional stability of aquatic ecosystems (Carpenter and Lodge, 1986; Scheffer et al., 1992). Their habitat structure, reproductive system, and gene flow are quite different from those of terrestrial plants (Barrett et al., 1993; Van Zuidam and Peeters, 2015). Generally, morphological plasticity is important for submerged macrophytes to adapt to a changing water environment (Barrett et al., 1993; Strand and Weisner, 2001). Submerged macrophytes can adapt to fluctuations of the water level or low-light stress by changing their stem length, number of branches, aboveground and belowground biomass allocation, and leaf area index (Strand and Weisner, 2001; Zhu et al., 2012; Yuan et al., 2016). Morphological plasticity is closely related to the ecological stoichiometric characteristics and stoichiometric homeostasis of submerged macrophytes (Li et al., 2015; Leal et al., 2017). The ability of submerged macrophytes to adjust their own chemical elements and the proportions of the elements is of great importance for adapting to the changing environments (Sistla and Schimel, 2012).

P is a limiting element in many aquatic ecosystems, and excessive P input leads to cyanobacterial bloom, thus triggering ecological imbalances and a series of environmental problems (Elser and Bennett, 2011; Tong et al., 2017). Submerged macrophytes have a strong ability to absorb P (Zhang et al., 2011; Christiansen et al., 2016). Previous field investigations of Güsewell and Koerselman (2002), Su et al. (2016), and our group (Li et al., 2013, 2017) showed that the P content of aquatic plants was more affected by the nutrient levels in the environment than by interspecific variations, suggesting that the P content of aquatic plants may be closely related to plant survival strategies and environmental adaptability. In addition, according to the growth rate hypothesis (GRH) in the theory of ecological stoichiometry (Elser et al., 2000; Sterner and Elser, 2002), organisms with a high growth rate have a high P content and low carbon (C): P and N: P ratios in their tissues, because the P content of ribosomal RNA is high and ribosomal RNA is closely related to the growth rate. The GRH has been verified in many studies (Lovelock et al., 2007; Yu et al., 2012; Xing et al., 2016). However, the degree to which submerged macrophytes maintain their tissue P contents in various nutrient levels (P stoichiometric homeostasis) and the corresponding influencing factors are still not very clear.

In this study, the stoichiometric characteristics and homeostasis of P in the aboveground and belowground parts of three submerged macrophytes with great differences in growth form were studied under different growth times and nutrient levels via laboratory experiments. The following questions were explored: (1) the characteristics of the P stoichiometric homeostasis of submerged macrophytes and the corresponding interspecific difference; and (2) the main factors affecting the P stoichiometric homeostasis of submerged macrophytes. This study is of great significance for understanding the P stoichiometric characteristics in submerged macrophytes, which provides a scientific basis for screening the pioneer species in the ecological restoration of polluted water.

## Materials and Methods

### Species Selection and Pretreatment

*Vallisneria natans* (Lour.) Hara, *Hydrilla verticillata* (L. f.) Royle and *Ceratophyllum demersum* (L.), which are very common in the waters of China, were the studied macrophyte species. The growth forms and biomass allocation strategies of these three submerged macrophytes are very different. *C. demersum* is a canopy-type submerged macrophyte, which can concentrate most of its biomass on the surface of the water to obtain maximum light. Because *C. demersum* does not have roots, it relies on the lower part of the stems and leaves buried in the sediment to attach the macrophyte to the substrate. Asexual reproduction often occurs by breaking off stems (Best, 1980; Gross et al., 2003). *V. natans* is a rosette-type submerged macrophyte, with long striped leaves, well-developed roots, and short upright stems (Xie et al., 2005; Fu et al., 2012). *H. verticillata* is an erect-type submerged macrophyte, whose biomass allocations to leaves, stems, and roots are relatively even (Langeland, 1996). The biomass ratios of the aboveground and belowground parts of these three macrophytes are in the order of *C. demersum* > *H. verticillata* > *V. natans*, and the macrophytes may have different nutrient absorption and metabolism strategies. Although different in many ways, the three studied species often coexist in many submerged macrophyte communities.

The three studied species of submerged macrophytes were all collected in Poyang Lake. *C. demersum* and *H. verticillata* were grown by apical shoots, and *V. natans* was grown by seedlings with roots according their growth characteristics. To get a uniform initial growth condition, the apical shoots or seedlings were selected to have the same size, with normal growth, and no branches. The length of the apical shoot was 15 cm, and the macrophyte height and root length of the *V. natans* seedlings were 12 and 3 cm, respectively. In order to simulate natural growth condition, all the three macrophyte species are grown together.

### Experimental Design

This experiment was carried out in 12 medium-sized glass tanks (0.6 m × 0.5 m × 1.0 m) at the Poyang Lake Model Test Research Base (115° 50′ 14.98″ N, 29° 13′ 19.57″ E). The glass tanks were put in a large greenhouse (180 m in length, 110 m in width, and 21 m in height) whose roof was made of steel frame with large panes of glass, which was very helpful to control the light intensity.

Ceramic sands with diameter of 1 ± 0.5 mm were bought and used as the substrate to fix the submerged macrophytes in this study. The pretreated apical shoots or seedlings of the three selected submerged macrophytes were planted in plastic square trays (19.5 cm × 13.5 cm × 5 cm) containing a 4-cm thick layer of substrate. Each tray was used to plant four individuals of one species, and each glass tank contained six trays, consisting of two trays each of the three species. These submerged macrophytes were subjected to the experimental treatment after 7 days of adaptive growth. Air aerated tap water was added to the glass tank to a depth of 0.8 m and a total volume of 240 L. The concentrations of total dissolved N and total dissolved P in the tap water were 0.02 ± 0.01 and 0.01 ± 0.00 mg/L, respectively. Different amounts of ammonium sulfate solution and monopotassium phosphate solution were added to each glass tank, and each treatment was performed in triplicate. The nutrient enrichment treatments were CK, for which no extra nutrient was added into the glass tank, T1, for which 48 mg N and 24 mg P were added, making 0.2 mg/L N content and 0.1 mg/L P contents in the water column in the beginning of the experiment, T2, for which 96 mg N and 48 mg P were added, making 0.4 mg/L N content and 0.2 mg/L P contents in the water column in the beginning of the experiment, and T3, for which 192 mg N and 96 mg P were added, making 0.8 mg/L N content and 0.4 mg/L P contents in the water column in the beginning of the experiment. The nutrient solutions were added once a week and the physicochemical indicators of water were determined. Tap water was added into the glass tanks to maintain all of them 240 L of water.

### Sample Processing and Parameter Measuring

The experiment was carried out for 40 days, and macrophyte sampling was performed on the 20th and 40th days, respectively. On macrophyte sampling days, one tray of each species in each glass tank was taken out and all macrophyte biomass in the tray was divided into aboveground and belowground parts. The samples were washed carefully with deionized water and processed immediately. They were repeatedly dried with water-absorbing paper until no water dropped by hard shaking. The fresh weight of the submerged macrophytes was determined by electronic balance. After weighing, the macrophyte samples were desiccated at 105°C for 1 h then dried at 70°C to constant weight, and ground to a uniform fine powder. The P content of the macrophyte was determined using a sulfuric acid-hydrogen peroxide digestion and ammonium molybdate – antimony potassium tartrate – ascorbic acid spectrophotometric method (Kuo, 1996).

During the experiment, water temperature (T), dissolved oxygen (DO), conductivity (COND), total dissolved solids (TDS), oxidation-reduction potential (ORP), and chlorophyll content (Chl) were measured using a handheld multi-parameter water quality meter (HQ40D, Hach Inc., United States), and total dissolved N (TDN) and total dissolved P (TDP) were measured using a standard method (Huang, 2000).

### Statistical Analysis

The relative growth rate (RGR) of the submerged macrophytes was calculated using the equation RGR = ln(*M*_2_/*M*_1_)/d*t*, where *M*_1_ is the initial fresh weight of the submerged macrophyte, *M*_2_ is the fresh weight of the submerged macrophyte after sampling, and d*t* is the growth days of the submerged macrophyte.

According to the principle of ecological stoichiometry (Sterner and Elser, 2002), the stoichiometric homeostasis of an element in a plant refers to the capacity of the element to be stable in a changing environment. It can be expressed by the stoichiometric homeostasis index *H* calculated by the formula *y* = *cx*^1/^*^H^*, where *y* is the content of an element or the element stoichiometric ratio in the plant, *x* is the content of the element or the stoichiometric ratio in the external environment, and *c* is a constant. The formula can be converted to log*y* = log*c*+1/*H*log*x*, and *H* can be obtained according to the regression relationship between log *x* and log y. According to Persson et al. (2010), the stoichiometric homeostasis of a species can be classified as follows: plastic (0 < *H* < 1.33), weakly plastic (1.33 < 1/*H* < 2), weakly homeostatic (2 < *H* < 4), and homeostatic (*H* > 4).

Data processing, analysis and plotting were completed using SPSS software (SPSS V16.0, SPSS Inc., Chicago, IL, United States). The differences between treatments on the physicochemical parameters, macrophyte morphologies and physiological parameters were analyzed by the one-way analysis of variance (ANOVA) method. The effects of growth parts (aboveground parts, belowground parts), growth length (20 days, 40 days), and nutrient levels (CK, T1, T2, and T3) on P contents of submerged macrophytes were analyzed by three-way ANOVA.

We used variance partitioning based on the sum of squares (SS) of three-way ANOVA with growth parts (P), growth length (L), and nutrient levels (N) as factors to indicate their contribution to the variance in the P concentrations (Güsewell and Koerselman, 2002; Li et al., 2013). The total SS of the ANOVA was decomposed as: SS_total_ = SS_P_ + SS_T_ + SS_N_ + SS_Error_. Variance contribution of each factor was then expressed as percentage of total SS (SS%).

## Results

### Characteristics of Water Quality Parameters

During the experiment, the temperature of the water body was about 32°C. In the treatment groups (T1–T3), the increasing level of nutrients led to different degrees of growth of the algae in the water column, thus causing an increase in chlorophyll content. The DO in the water column was in a supersaturated state (over 100%). With the increasing amount of added nutrients, TN, TP, COND, TDS, DO, and Chl contents increased significantly, while ORP showed an upward trend (**Table 1**).

**Table 1:** Physic-chemical characteristics of the water at different treatments.

	CK	T1	T2	T3
T(°C)	31.53(0.14)^a^	31.67(0.30)^a^	31.60(0.11)^a^	31.79(0.22)^a^
COND (μS/cm)	96.30(1.02)^a^	119.95(16.67)^b^	133.63(19.14)^bc^	146.53(12.43)^c^
TDS (mg/l)	55.50(0.58)^a^	69.25(9.54)^b^	77.00(10.92)^bc^	84.25(6.95)^c^
DO (% sat)	121.70(2.89)^a^	192.05(27.21)^b^	209.45(16.21)^b^	235.28(10.58)^c^
ORP (mV)	95.48(2.13)^a^	132.08(7.31)^b^	139.23(10.35)^bc^	145.33(4.72)^c^
Chl (μg/L)	0.12(0.07)^a^	33.39(28.74)^ab^	51.10(23.65)^b^	145.31(48.20)^c^
TDN (mg/L)	0.068(0.023)^a^	0.390(0.022)^b^	0.748(0.025)^c^	1.423(0.048)^d^
TDP (mg/L)	0.029(0.004)^a^	0.129(0.006)^b^	0.232(0.008)^c^	0.423(0.010)^d^


*Values are shown as mean (SD). Different letters on the top right of the values indicate significant difference at the 0.05 level.*

### Growth Characteristics of Submerged Macrophytes

**FIGURE 1:**
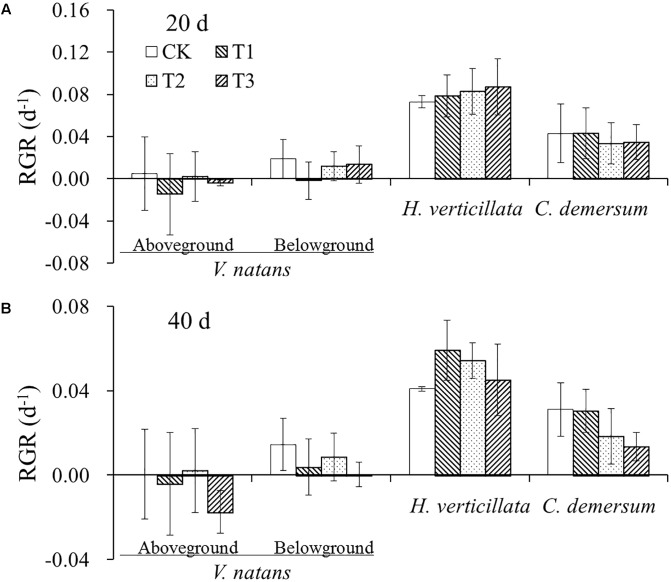
The change of relative growth rates (RGR) of the three studied submerged macrophytes in **(A)** 20 days and **(B)** 40 days in response to different nutrient treatments.

The average initial weights of the aboveground and belowground parts of *V. natans* were 0.79 ± 0.17 g and 0.23 ± 0.04 g, respectively. The growth of *V. natans* was slow, and the average growth rates were close to zero in 20 and 40 days. The initial weight of the aboveground part of *H. verticillata* was 0.32 ± 0.06 g. The average growth rates of the aboveground part were 0.08 ± 0.02 and 0.05 ± 0.01 d^-1^, respectively, in 20 and 40 days. The initial weight of the aboveground part of *C. demersum* was 0.59 ± 0.19 g. The average growth rates of the aboveground part were 0.04 ± 0.02 and 0.02 ± 0.01 d^-1^, respectively, in 20 and 40 days. The growth rate of *C. demersum* was faster than that of *V. natans* and slower than that of *H. verticillata*. There were also no significant differences in the growth rate of the aboveground and belowground parts among the three treatments and CK groups in the two growth stages for all the three species (**Figure 1**).

### P Contents and Stoichiometric Homeostasis of Submerged Macrophytes

**FIGURE 2:**
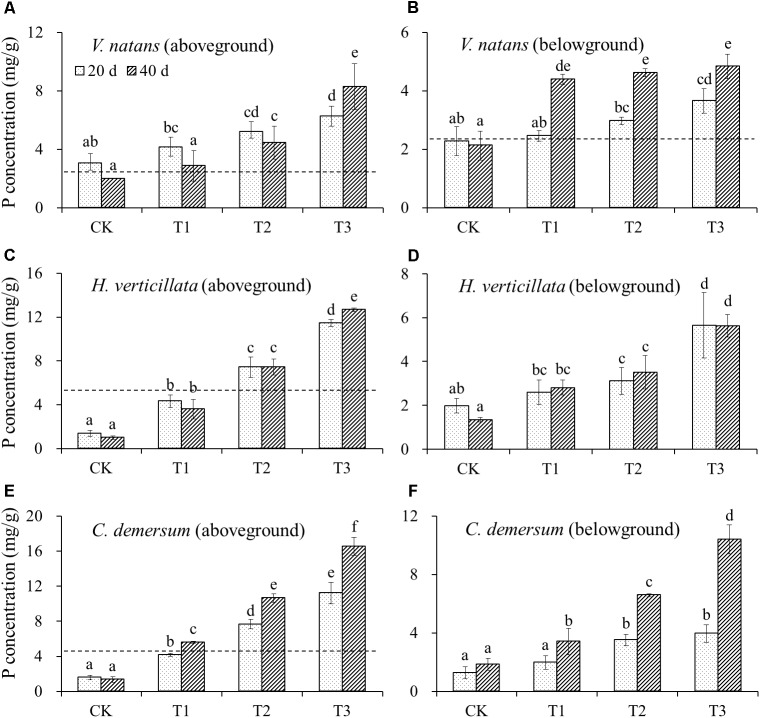
The change of P contents in **(A)** aboveground of *V. natans*, **(B)** belowground of *V. natans*, **(C)** aboveground of *H. verticillata*, **(D)** belowground of *H. verticillata*, **(E)** aboveground of *C. demersum*, and **(F)** belowground of *C. demersum* in response of growth time and nutrient treatments. Dash lines represent initial P contents of the studied macrophytes before planted. Different letters indicate significant difference at the 0.05 level.

The initial P content in the aboveground part of *V. natans* was 2.60 ± 0.25 mg/g, and the P contents in the aboveground part of the treatment groups and CK groups on the 20th day were higher than the initial levels. In the aboveground part on the 40th day, except for the CK groups whose P content was lower than the initial level, all of the treatment groups showed an increase in the P contents. The initial P content of the belowground part of *V. natans* was 2.41 ± 0.10 mg/g, the P contents of the CK groups on the 20th and 40th days were lower than the initial levels, but the P contents of the treatment groups were all higher than the initial levels. The P contents of the aboveground and belowground parts of *V. natans* increased significantly with the increasing P content in the water body (**Figure 2**).

The initial P content in the aboveground part of *H. verticillata* was 5.14 ± 0.20 mg/g, and the P contents in the CK and T1 groups decreased on the 20th and 40th days, while the P contents of the T2 and T3 groups increased. The P contents of the aboveground and belowground parts of *H. verticillata* increased significantly with the increasing P content in the water body (**Figure 2**).

The initial P content in the aboveground part of *C. demersum* was 4.63 ± 0.56 mg/g, the P contents of the CK and T1 groups on the 20th day were lower than the initial levels, and the P contents of the T2 and T3 groups were higher than the initial levels. The P content of the aboveground part of *C. demersum* was significantly increased with the increasing P content in the water body, and the P content of the belowground part increased slightly on the 20^th^ day but increased substantially on the 40th day (**Figure 2**).

**FIGURE 3:**
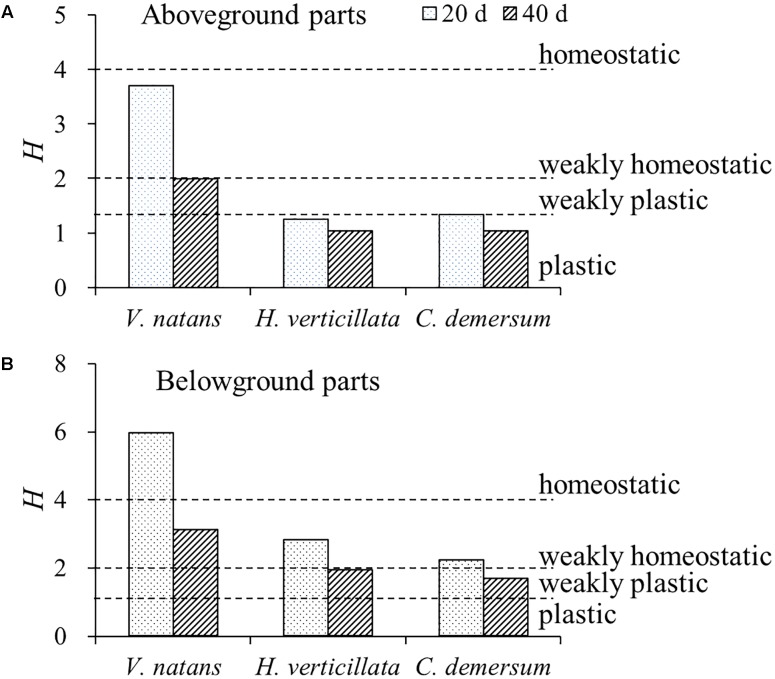
Stoichiometric homeostasis index (*H*) of **(A)** aboveground and **(B)** belowground parts of the three submerged macrophytes in 20 and 40 days. The *H*-values were calculated from the equation of linear regression between LN-transformed phosphorus contents in water and in submerged macrophytes. Dash lines represent threshold values of *H* classified as follows: plastic (0 < *H* < 1.33), weakly plastic (1.33 < 1/*H* < 2), weakly homeostatic (2 < *H* < 4), and homeostatic (*H* > 4), according to Persson et al. (2010).

For the P stoichiometric homeostasis index (*H*_P_), the values of the aboveground parts of the three submerged macrophytes were smaller than those of the belowground parts, the values on the 20th day were higher than those on the 40th day, the value of *V. natans* was higher than those of *H. verticillata* and *C. demersum*, and no significant difference (*p* > 0.05) was found in this value between *H. verticillata* and *C. demersum* (**Figure 3**). All of the aboveground parts of the three submerged macrophytes exhibited plastic or weakly plastic P stoichiometric homeostasis except for *V. natans*. However, all of the belowground parts of the three submerged macrophytes exhibited weakly homeostatic or homeostatic P stoichiometric homeostasis in 20 days growth (**Figure 3**).

### Factors Affecting P Contents in the Submerged Macrophytes

The organ and nutrient level had a significant effect on the P contents of *V. natans*, *H. verticillata*, and *C. demersum*, while the growth length only had a significant effect on the P content in *C. demersum* (**Table 2**). Among the three factors, the nutrient level had the greatest effect on the P content in the submerged macrophytes, and the variance contribution rate was over 50%. The organ had the second greatest impact on the P content, and the variance contribution rate was between 10 and 20%. The growth length had no significant effect on the P contents of *V. natans* and *H. verticillata*, but its variance contribution rate to the P content of *C. demersum* was 9.16%.

**Table 2:** Effects of growth parts (aboveground parts, belowground parts), growth length (20 days, 40 days), and nutrient levels (CK, T1, T2, and T3) on P contents of submerged macrophytes.

Source	SS	df	F	Sig.	SS%
*V. natans*					
Growth part	19.67	1	15.53	0.000	10.09
Growth length	3.16	1	2.50	0.120	1.62
Nutrient level	98.67	3	25.97	0.000	50.61
Error	73.45	58			
*H. verticillata*					
Growth part	128.03	1	54.23	0.000	17.09
Growth length	0.00	1	0.00	0.979	0.00
Nutrient level	484.06	3	68.34	0.000	64.62
Error	136.95	58			
*C. demersum*					
Growth part	165.97	1	50.73	0.000	13.70
Growth length	110.94	1	33.91	0.000	9.16
Nutrient level	744.86	3	75.90	0.000	61.48
Error	189.74	58			


*SS: Type III Sum of Squares; SS%: the relative variance rate; df: degree of freedom.*

## Discussion

In this study, the characteristics of P content in three submerged macrophytes with different growth forms, different growth rates, and different characteristics of biomass allocation were studied. The results showed that the responses of the three submerged macrophytes to the increasing nutrient levels in the water were consistent. That is, the P content for all of the macrophytes increased with the increasing nutrient contents in the water body. However, the three submerged macrophytes exhibited significant differences on the P stoichiometric homeostasis. The *H*_P_ of *V. natans* was significantly higher than those of *H. verticillata* and *C. demersum*, and no significant difference was found between *H. verticillata* and *C. demersum*. The study by Xing et al. (2016) found that the N:*H*_P_ of *V. natans* was significantly higher than those of *C. demersum* and *Myriophyllum spicatum*, suggesting that the high level of stoichiometric homeostasis might be an essential feature of *V. natans*. The study by Yu et al. (2010) on the vascular plants in the grassland of Inner Mongolia showed that the plants with a high level of stoichiometric homeostasis had a relatively high productivity and community stability. This is because plants with a high level of stoichiometric homeostasis are more conservative on nutrient absorption and utilization, so they are dominant in low-nutrient ecosystems. In the ecosystems with a high nutrient level, the submerged macrophytes are under high nutrient stress. The submerged macrophytes with a low level of stoichiometric homeostasis can absorb and store more nutrients, but consume more hydrocarbons at the same time (Cao et al., 2011; Yuan et al., 2013), which may be unfavorable for the reproduction of submerged macrophytes and resistance to environmental interference.

This study indicated that the species, organs, growth length, and nutrient level could significantly affect the P content of the submerged macrophytes, and the nutrient level had the greatest impact, followed by the organs, while the impact of species and growth length were relatively small. This finding confirmed the author’s previous field study that the P content of submerged macrophytes was more affected by the environment than by interspecific variations (Li et al., 2017) and it was consistent with other wetland plants (Güsewell and Koerselman, 2002), indicating that the P content in the plants could effectively indicate the availability of P in the environment. Additionally, the results of this study showed that submerged macrophytes with higher P contents exhibited a lower level of P stoichiometric homeostasis, which indicated that the characteristics of P stoichiometric homeostasis of submerged macrophytes were consistent with that of terrestrial plants (Yu et al., 2011). There is a phenomenon of luxury consumption for submerged macrophytes in a high-P environment. Furthermore, the P contents and growth rates of *H. verticillata* and *C. demersum* were higher than that of *V. natans* in this study, suggesting that the P content of submerged macrophytes was consistent with the change in their growth rate, which is in accordance with the GRH.

The average water temperature in this study was over 30°C. Therefore, with the increasing nutrient content in water body, the growth rate of algae in the water body also increased, which was manifested by significant increases in the conductivity, turbidity, and chlorophyll content of the water. Submerged macrophytes grown in high levels of nutrients may be subjected to severe low-light stress, which might be one of the reasons why no significant differences were found in the biomass and growth rate of the submerged macrophytes among treatments. However, the P contents of the aboveground and belowground parts of the three submerged macrophytes increased with the increasing nutrient concentration in the water body, and the P content of *H. verticillata* and *C. demersum* in the T3 treatment group was seven- to eightfold higher than that of the CK group. The above results suggested that although the high nutrient stress affected the growth rate of the submerged macrophytes, it had a minor impact on P absorption by the submerged macrophytes, especially for *H. verticillata* and *C. demersum*. In addition, the results in our study which conducted in the laboratory are not very consistent with results in field conditions. For example, our previous field study (Li et al., 2017) indicated that P contents of water in Dianchi Lake were four times as much as those in Erhai Lake, while P contents of submerged macrophytes in Dianchi Lake were not significantly different from those in Erhai Lake. These differences may be mainly resulted from the length of growth time for submerged macrophytes. Greenhouse experiments often conducted in short period which often last from several hours to less than one year, while in field long-term observation the second-year growth of plants was usually affected by their nutrient storage in previous year (Güsewell, 2005; Li et al., 2015).

Submerged macrophytes can absorb the N and P not only in the sediment through their belowground parts, but also in the water body through their aboveground parts (Rattray et al., 1991). When the N and P concentrations in the water are higher than the requirement of the submerged macrophytes, they can absorb excessive N and P and store them in tissues (Xing et al., 2016). Therefore, the N and P contents of submerged macrophytes are highly plastic, reflecting the N and P nutritional status of the aquatic environment in which they are present, and they can serve as the quality indicators for the water environment. Previous studies (Li et al., 2015, 2017) showed that the variation coefficient of the P content in submerged macrophytes is greater than that of N, and that P is a limiting element for many water bodies. An increase in P content is one of the important reasons for cyanobacterial bloom (Tong et al., 2017). Therefore, monitoring the P content of submerged macrophytes is crucial for understanding the bioavailability of P. In this study, the levels of P stoichiometric homeostasis of *H. verticillata* and *C. demersum* were significantly lower than that of *V. natans*, so *H. verticillata* and *C. demersum* were ideal indicator organisms for the bioavailability of P in the water body. *H. verticillata* showed the highest growth rate among the three submerged macrophytes and a higher accumulation of P, which might be the most suitable species for removing P from the water body in a short period of time.

## Author Contributions

WL, YL, and HF contributed to the conception and design of the study. WL, YL, JZ, HF, JT, and HbF performed the experiments and the statistical analysis. WL wrote the manuscript. All the authors contributed to the manuscript revision, read and approved the submitted version.

## Conflict of Interest Statement

The authors declare that the research was conducted in the absence of any commercial or financial relationships that could be construed as a potential conflict of interest.
